# Supplementary material for: Three-Dimensional Modeling with Osteoblast-like Cells under External Magnetic Field Conditions Using Magnetic Nano-Ferrite Particles for the Development of Cell-Derived Artificial Bone
Source: Nanomaterials (Basel). 2024 Jan 23;14(3):251. doi: 10.3390/nano14030251 (PMC10857141; doi:10.3390/nano14030251)
Supplement: Supplementary file 1 [file nanomaterials-14-00251-s001.zip › nanomaterials-2830092-Supplementary Information-done.pdf]

nanomaterials

Supplementary Information

Three-Dimensional Modeling with Osteoblast-Like Cells under External Magnetic Field Conditions Using Magnetic Nano-Ferrite Particles for the Development of Cell-Derived Artificial Bone

Chuang Ma [1,2], Makoto Izumiya [1,2], Hidehiko Nobuoka [1,3], Rintaro Ueno [1,3], Masaki Mimura [1,3], Katsuya Ueda [1,2], Haruka Ishida [1,2], Daihachiro Tomotsune [1,4], Kohei Johkura [4], Fengming Yue [1,4], Naoto Saito [1] and Hisao Haniu [1,2,3,*]

[1] Institute for Biomedical Sciences, Interdisciplinary Cluster for Cutting Edge Research, Shinshu University, 3-1-1 Asahi, Matsumoto, Nagano 390-8621, Japan
[2] Biomedical Engineering Division, Graduate School of Medicine, Science and Technology, Shinshu University, 3-1-1 Asahi, Matsumoto, Nagano 390-8621, Japan
[3] Biomedical Engineering Division, Graduate School of Science and Technology, Shinshu University, 3-1-1 Asahi, Matsumoto, Nagano 390-8621, Japan
[4] Department of Histology and Embryology, School of Medicine, Shinshu University, 3-1-1 Asahi, Matsumoto, Nagano 390-8621, Japan
* Correspondence: hhaniu@shinshu-u.ac.jp; Tel.: +81-263-37-3555

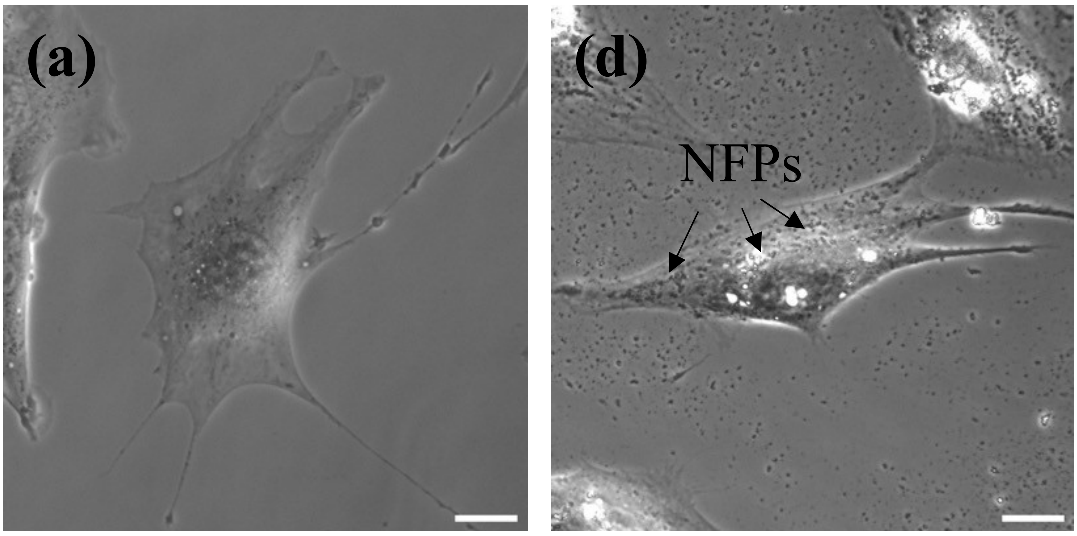

Figure S1. Phase contrast illumination image of MC3T3-E1 cells. **(a)** MC3T3-E1 cells control group culture without NFPs. **(b)** MC3T3-E1 cells exposed to 0.14 mg/mL NFPs for 72 h. Arrows indicate NFPs. Scale bar: 5 μm.

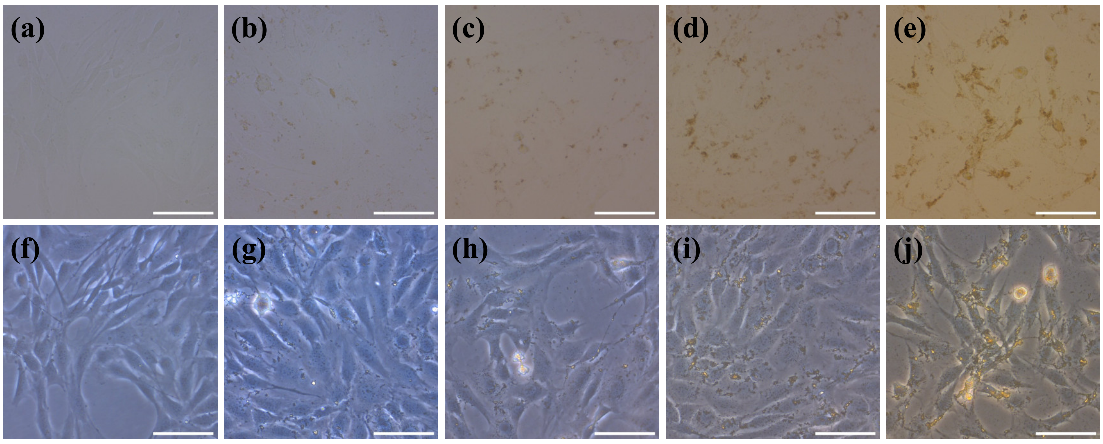

Figure S2. Cellular uptake of NFPs in MC3T3-E1 cells. **(a)** Bright-field image of MC3T3-E1 cells control group without NFPs. **(b–e)** Bright-field image of MC3T3-E1 cells culture exposed to 0.07 to 0.57 mg/mL NFPs for 72 h. **(f)** Phase contrast illumination image of MC3T3-E1 cells control group without NFPs. **(g–j)** Phase contrast illumination image of MC3T3-E1 cells culture exposed to 0.07 to 0.57 mg/mL NFPs for 72 h. Scale bar: 50 μm.
